# Depression among Parents Two to Six Years Following the Loss of a Child by Suicide: A Novel Prediction Model

**DOI:** 10.1371/journal.pone.0164091

**Published:** 2016-10-03

**Authors:** Tommy Nyberg, Ida Hed Myrberg, Pernilla Omerov, Gunnar Steineck, Ullakarin Nyberg

**Affiliations:** 1 Division of Clinical Cancer Epidemiology, Department of Oncology-Pathology, Karolinska Institutet, Stockholm, Sweden; 2 Childhood Cancer Research Unit, Department of Women’s and Children’s Health, Karolinska Institutet, Stockholm, Sweden; 3 Department of Health Care Sciences, Ersta Sköndal University College, Stockholm, Sweden; 4 Division of Clinical Cancer Epidemiology, Department of Oncology, Institute of Clinical Sciences, Sahlgrenska Academy at the University of Gothenburg, Gothenburg, Sweden; 5 Stockholm Centre for Psychiatric Research and Education, Department of Clinical Neuroscience, Karolinska Institutet, Stockholm, Sweden; Centre Hospitalier Universitaire Vaudois, FRANCE

## Abstract

**Background:**

Parents who lose a child by suicide have elevated risks of depression. No clinical prediction tools exist to identify which suicide-bereaved parents will be particularly vulnerable; we aimed to create a prediction model for long-term depression for this purpose.

**Method:**

During 2009 and 2010 we collected data using a nationwide study-specific questionnaire among parents in Sweden who had lost a child aged 15-30 by suicide in years 2004-2007. Current depression was assessed with the Patient Health Questionnaire (PHQ-9) and a single question on antidepressant use. We considered 26 potential predictors assumed clinically assessable at the time of loss, including socio-economics, relationship status, history of psychological stress and morbidity, and suicide-related circumstances. We developed a novel prediction model using logistic regression with all subsets selection and stratified cross-validation. The model was assessed for classification performance and calibration, overall and stratified by time since loss.

**Results:**

In total 666/915 (73%) participated. The model showed acceptable classification performance (adjusted area under the curve [AUC] = 0.720, 95% confidence interval [CI] 0.673-0.766), but performed classification best for those at shortest time since loss. Agreement between model-predicted and observed risks was fair, but with a tendency for underestimation and overestimation for individuals with shortest and longest time since loss, respectively. The identified predictors include female sex (odds ratio [OR] = 1.84); sick-leave (OR = 2.81) or unemployment (OR = 1.64); psychological premorbidity debuting during the last 10 years, before loss (OR = 3.64), or more than 10 years ago (OR = 4.96); suicide in biological relatives (OR = 1.54); with non-legal guardianship during the child’s upbringing (OR = 0.48); and non-biological parenthood (OR = 0.22) found as protective.

**Conclusions:**

Our prediction model shows promising internal validity, but should be externally validated before application. Psychological premorbidity seems to be a prominent predictor of long-term depression among suicide-bereaved parents, and thus important for healthcare providers to assess.

## Introduction

The loss of a child by suicide is a severe trauma that is associated with higher risks of long-term psychological morbidity as compared to parents from the general population [1–4], and parents bereaved from non-violent causes [1, 4–7]. Suicide-bereaved parents have a higher suicide risk [8] and overall mortality [9].

Depression is a common complication following bereavement from unnatural causes [1, 6] and recovery trajectories seem to be longer than after bereavement from natural causes [6]. Brief episodes of depression immediately post-loss are often less severe than long-lasting bereavement-related depression [10]. When planning an intervention in a trauma-struck cohort, it is important to be able to identify individuals who are at the highest risk of long-term morbidity and who consequently could have the greatest potential gain from a successful pre- or postmorbidity intervention early in the grief process. Bereavement-related depression may be effectively treated [11], and early detection of depressive symptoms has been reported to enhance the success rates of depression treatment [12].

Apprehensions about conducting studies regarding bereavement after suicide are common, possibly because of the taboos and stigmas surrounding suicide [13]. Ethics committees require evidence that the proposed study will not cause participants distress or enhance suicidal ideation, but there are few studies addressing these concerns [14–16]. From register-based research and small sample questionnaire and interview studies on groups of bereaved who have lost a next-of-kin to suicide, a number of risk factors have been suggested for psychological morbidity and proxies such as psychiatric admission or subsequent death by suicide. These include general predictors of morbidity such as sex, age and socio-economics [7, 17, 18], history of psychological morbidity [8, 17], suicide-specific predictors such as prior suicide attempts in the deceased and exposure to the dead body [19], and biological or social predisposition as indicated by familial patterns in psychological morbidity and suicide [4, 8, 20].

Statistical regression modeling techniques represent a structured way of considering the impact of multiple potential predictors on an outcome. Medical practitioners sometimes have difficulties in interpreting the results of such models; in a 2006 US survey among medical residents, only 37% of participants could correctly interpret the output from a logistic regression model [21]. To overcome this, Harrell [22] has advocated the creation and use of reference nomograms according to the methods originally devised by Banks [23] to visualize associations between model predictors and outcome, and to give the end user not only a measure of association for each model predictor but also an easily used tool to calculate model-predicted probabilities for individual subjects.

Bereavement intervention programs are more efficient when targeted at vulnerable individuals than when offered to all bereaved [5]. Given valid prediction tools, interventions may be directed at high-risk individuals. Prediction models for depression have been proposed both for parents bereaved by suicide [18], and any cause [24]. However, the clinical utility of these models are limited by their inclusion of predictors which may be difficult to clinically assess at the time of loss, e.g. personality features [18], or post-loss circumstances unknown at time of loss, e.g. help-seeking behavior [18, 24] and complicated grief and suicidality [18].

The aim of this study was to create a prediction model which could be applied at the time of loss to assess the risk of long-term depression among suicide-bereaved parents. Most suicide-bereaved parents come into contact with health services pertaining to the death of their child, but to our knowledge no prediction tools are available to identify individuals at high risk of long-term psychological morbidity, based on clinically assessable information at the time of loss. With this in mind, we have used data from a questionnaire collected nationwide among parents who have lost a son or daughter by suicide, to construct a novel prediction model where we took special care to only consider potential predictors that can be assumed to be known at the time of loss and easy for healthcare personnel to ask about or otherwise assess.

## Materials and Methods

### Data collection and ethical considerations

We identified all individuals in Sweden who died by suicide at ages 15 to 30 between the years 2004 and 2007, as registered in the Swedish Cause of Death Register (ICD-10: X60-X84). The bereaved parents of the deceased were identified through the national Multigeneration Register. Inclusion criteria comprised being born in one of the Nordic countries, ability to communicate in Swedish, and having an identifiable address and telephone number. Parents who had lost more than one child were not included.

We have previously described the data collection and ethical protocol followed throughout the study [16, 25]. In brief, we developed a study-specific questionnaire based on interviews with 17 suicide-bereaved parents. This preliminary questionnaire was subsequently validated with a total of 46 suicide-bereaved parents, after which we made minor adjustments to ensure that the questions were correctly understood and not upsetting to the participants. The final questionnaire contains 175 questions, and covers the parent’s current situation, well-being and history of psychological morbidity, the relationship between parent and child, the time before the child’s suicide including psychological morbidity in the child, the suicide and related circumstances, and the time following the suicide including support measures. We used this questionnaire to anonymously collect all data during 2009 and 2010, two to six years after the loss. We obtained informed oral consent through telephone, which was noted in our database and confirmed by a returned and completed questionnaire. For ethical reasons, we did not obtain written consent during contact as we did not want the parents to feel pressured to complete participation. The study as well as our contact and consent procedures was approved by the Regional Ethical Review Board in Stockholm, Sweden.

### Outcome and considered potential predictors

We measured depression with the 9-item depression subscale of the Patient Health Questionnaire (PHQ-9), using PHQ-9 sum ≥ 10 to indicate moderate to severe depression [26]. In order to avoid misclassification of those with a managed clinical depression at the time of questionnaire response, those with lower PHQ-9 sums but ongoing use of anti-depressant medication (≥ 1 dose per week) were also categorized as depressed.

To reduce the risk of misclassification related to current psychological mood affecting perception of previous events, we chose to focus on variables which describe formal circumstances known at the time of loss as recall-related issues may be less significant for such circumstances [27]. We use the term “predictors” in a loose sense as variables associated, not necessarily causally, with risk of depression and which can aid in its prediction [28]. In total we considered 26 potential predictors of depression, including socio-economic background parameters of parent and child [7, 8, 18], the relationship between parent and child [19, 29], history of psychological stress and morbidity in parent and child [2, 8, 29], and formal circumstances related to the child’s suicide [19]. The variables considered were mostly asked as closed-ended multiple choice questions.

The parents were asked about civic and employment status both at the time of loss and current, and we considered only the former as potential predictors. For employment status at the time of loss, we only asked whether the parent had been on sick-leave or without employment and classified the parents into three groups. Respondents without employment were handled according to their age at the time: those below the age of 65 were classified as unemployed, whereas those aged 65 or above, the legal age for retirement in Sweden, were classified as not on sick-leave nor unemployed.

We assessed the parents’ history of psychological morbidity with four questions regarding whether they had previously received: treatment for psychological problems; a psychiatric diagnosis; medication against anxiety; or, against low mood or depression. Each question had a follow-up question on time of first receipt, with response options: “more than 10 years earlier”, “during the last 10 years, before my child’s death” and “during the last 10 years, after my child’s death”. We classified parents who reported to have received any of the four treatments or diagnoses *before* their child’s death as having a history of psychological morbidity which debuted in the earliest reported of the two time-frames, whereas we treated parents with no morbidity or who had received treatment or diagnosis exclusively *after* the child’s death as having no psychological premorbidity at the time of loss.

We asked about formal circumstances surrounding the parents’ relationship with their child such as the frequency of contact and whether the child lived with the parent at the time of death. We also asked the parents to report the child’s history of contact with psychiatric services, as well as whether the child had had self-injurious behavior or had made previous suicide attempts. For circumstances surrounding the suicide, parents were asked about the suicide method; we divided the responses into poisoning or violent means. We asked how the parent had found out that their child had died and used this information to classify whether the parent had seen the body at the site of death or received the death notice later. We further recorded whether the parents had viewed their child’s body in at least one of four formal settings: the hospital, the hospital church, a forensic medicine department, or at the funeral parlor.

In addition, we asked a number of questions concerning the parents’ current worry for other family members, and subjective views of what might have triggered the suicide and if the suicide could have been prevented. We did not consider these variables as potential predictors, but exploratively assessed their associations with identified predictors post hoc to aid in the discussion on possible mechanisms.

The time since loss has been reported to affect bereavement-related outcomes such as depression, complicated grief and post-traumatic stress in suicide-bereaved groups [7, 18, 19, 30], and several studies have shown a decrease in depression risk with the time since loss in next-of-kin bereaved from other causes than suicide [24, 31, 32]. A preliminary analysis of our data showed an association between depression and time since loss, but since time since loss is not easily used as a predictor for a recently bereaved parent, we chose not to include it in the prediction model. To overcome the time-dependence of the outcome we have instead tested our model’s performance in various subsets according to the time since loss (see below).

### Statistical analysis

An expanded description of the creation of our multivariable prediction model is available in the supplementary S1 Appendix. In brief, we employed a nearest-neighbor imputation with Gower distance [33] and used the resulting imputation-completed data for all modeling, whereas we present the original incomplete data for all descriptives. We used logistic regression for all modeling, and present odds ratios (OR) with 95% confidence intervals (CI). Initially, we assessed non-linearity in the effect of continuous variables and selected one parametrization for each, and pairwise collinearity in our 26 considered potential predictors and selected only one variable to retain out of pairs of strongly correlated variables [34]. We then created the model, using all subsets selection with ten-fold stratified cross-validation (SCV) among multiple refolds of the data, with the aim of minimizing the model deviance. To reduce the number of possible models (and thus computational resources), we first considered only variables which had *p* < 0.25 in univariable logistic regression [35] and performed a preliminary all subsets selection, after which we reconsidered the initially omitted variables with univariable *p* ≥ 0.25 as well as interaction terms, before arriving at our final model (S1 Appendix).

We have visualized the model as a nomogram, where the ORs of the multivariable model were transformed and rescaled to a point-based scoring system in order to allow for easy calculation of model-predicted probabilities for individual bereaved parents. The lowest-risk categories of each variable were all assigned 0 points, while the category with the overall highest OR was assigned 100 points. Other categories were assigned between 0 and 100 points according to the quotient between their ORs in comparison with the lowest-risk category, and the overall highest OR [23, 36]. We assessed presence of multicollinearity in the model using variance inflation factors [37]. As measure of overall model fit we give McFadden’s R^2^. We assessed the classification performance, i.e. ability to accurately rank individuals from low to high probability of depression, with receiver operating characteristic (ROC) curves with the corresponding areas under the curve (AUC) as summary measure. AUC ranges from 0.5 (no discrimination) to 1.0 (perfect classification), and by the convention introduced by Hosmer & Lemeshow [35] values above 0.7, 0.8 and 0.9 are interpreted as indicating acceptable, excellent and outstanding discrimination, respectively. We give AUCs unadjusted and adjusted for overfitting using ten-fold SCV with 100 repetitions. CIs for the AUC were constructed using 2000 bootstrap resamples, and the confidence limits were in turn cross-validated. The 100 ROC curves were averaged using a LOESS fit. We assessed the model’s calibration, i.e. agreement between model-predicted and actual probability of depression, with calibration plots of model-predicted versus LOESS estimated risks.

Since depression was found to vary with time we divided the data into four equally-sized subsets according to time since loss (“time-frames”), and performed an internal-external validation [38] where we assessed classification performance in each time-frame based on a new model formed from individuals in the remaining three time-frames. We also assessed the model’s calibration in each time-frame.

Post hoc, we assessed associations between other questionnaire questions with the identified predictors using Goodman & Kruskal’s gamma correlation coefficient (*γ*). As sensitivity analysis, we omitted one of the identified predictors and repeated the model-building procedure in order to assess its exchangeability with our other considered predictors.

All calculations were performed with R software (version 3.2.2, R Foundation for Statistical Computing, Vienna, Austria) using the *rms*, *cluster*, *pROC* and *parallel* packages.

## Results

Out of 915 eligible parents, 666 (73%) agreed to participate and returned a questionnaire. Among those, one participant had failed to respond to both PHQ-9 and the question on antidepressant use and was excluded. Among the 665 included parents, 167 (25%) were classified as depressed, of which 47 both had a PHQ-9 sum ≥ 10 and used antidepressants at least once a week, 68 had a PHQ-9 sum ≥ 10 with no regular antidepressant use, and 52 regularly used anti-depressants without scoring 10 or above on PHQ-9. Pharmaceutical treatment of depression was thus not present in 68 among the 167 classified as depressed (41%).

The included participants were in median 52 years at loss (inter-quartile range 48-57). Fifty-seven percent were mothers, and 69% of the deceased were sons. Most had remaining children (93%), had been the child’s legal guardian (92%), and were biological parent (95%) (Table 1). Zero, one, two, and between three and nine missing values among the considered potential predictors, were seen in 581 (87.4%), 65 (9.8%), eight (1.2%), and eleven (1.7%) participants, respectively.

**Table 1:** Characteristics of the participating suicide-bereaved parents.

**Inclusion summary**	**n**	**(%)**
Eligible parents	915	
Non-participants	249	(27.2%)
Not reachable	8	(0.1%)
Declined participation	125	(13.7%)
Agreed but did not complete participation	116	(12.7%)
Participants	666	(72.8%)
Excluded due to missing information on depression status	1	(0.1%)
Included in present study	665	(72.7%)
**Characteristics of the included parents**	**n**	**(%)**
Sex		
Male	283	(42.6%)
Female	382	(57.4%)
Age at study participation		
Median [inter-quartile range]	56 [52-60]	
40-49	90	(13.5%)
50-64	517	(77.7%)
65-81	58	(8.7%)
Age at loss		
Median [inter-quartile range]	52 [48-57]	
35-49	211	(31.7%)
50-64	437	(65.7%)
65-75	17	(2.6%)
Family constellation at time of study		
Lives with a partner	477	(71.7%)
Has a partner but lives alone	44	(6.6%)
Widow/widower	18	(2.7%)
Single	120	(18.0%)
Not stated	6	(0.9%)
Lived with a partner at time of child’s death		
Yes	497	(74.7%)
No	164	(24.7%)
Not stated	4	(0.6%)
Residential area		
Rural	162	(24.4%)
Town (population <10,000)	152	(22.9%)
Small city (population <50,000)	128	(19.2%)
Mid-sized city (population <200,000)	117	(17.6%)
Larger city (population ≥200,000)	97	(14.6%)
Not stated	9	(1.4%)
Country of birth		
Sweden	628	(94.4%)
Other Nordic country	36	(5.4%)
Not stated	1	(0.2%)
Level of education		
Elementary school or less	146	(22.0%)
Secondary school	270	(40.6%)
University < 3 years	82	(12.3%)
University ≥ 3 years	159	(23.9%)
Not stated	8	(1.2%)
Source of income at time of study		
Employed or self-employed	498	(74.9%)
Old-age pension	58	(8.7%)
Disability pension	61	(9.2%)
Unemployment fund	25	(3.8%)
Study allowance	4	(0.6%)
Social security	3	(0.5%)
Other	9	(1.4%)
Not stated	7	(1.1%)
Remaining children following the loss		
Yes	618	(92.9%)
No	47	(7.1%)
Year of child’s death		
2004	162	(24.4%)
2005	173	(26.0%)
2006	169	(25.4%)
2007	161	(24.2%)
Sex of deceased child		
Male	461	(69.3%)
Female	204	(30.7%)
Age of deceased child		
Median [inter-quartile range]	20 [23-27]	
Legal guardian during child’s upbringing		
Yes	610	(91.7%)
No	54	(8.1%)
Not stated	1	(0.2%)
Biological parent		
Yes	634	(95.3%)
No	31	(4.7%)

### Model development

Descriptive frequencies and univariable ORs for the association between all 26 considered potential predictors and depression are shown in Table 2. Continuous predictors were assessed for non-linearity and the parametrizations identified subsequently used; however none were included in the final model. Three variable pairs were found to have pairwise correlation ≥ 0.5, yielding three variables omitted (omitted variable in brackets): [country of birth of parent], and of parent’s own parents; frequency of contact, and [whether the child lived with the parent at the time of the suicide]; and whether the child had made previous suicide-attempts during the last year alive, and [earlier than that]. Thus, a total of 23 variables remained as candidates for the multivariable model. Among those, 14 had univariable *p* < 0.25 and were used for the first all subsets selection. A preliminary model was identified which was subsequently retained as final model, as we found neither variables with *p* ≥ 0.25 nor interaction terms to improve the fit (see S1 Appendix).

**Table 2:** Univariable odds ratios for all considered potential predictors of long-term depression.

Variable	n/N	(%)	OR	(95% CI)	*p*-value
**Parent’s background**					
Sex					<0.001
Male	46/283	(16.3%)	1.00		
Female	121/382	(31.7%)	2.39	(1.63-3.50)	
Age at loss					0.076
per 5 years (continuous)^a^			0.88	(0.76-1.01)	
Country of birth^b^					0.987
Sweden	158/628	(25.2%)	1.00		
Other Nordic country	9/36	(25.0%)	0.99	(0.46-2.16)	
Country of birth of participant’s parents					0.307
Sweden	143/587	(24.4%)	1.00		
Other	23/77	(29.9%)	1.31	(0.78-2.22)	
Remaining children following the loss					0.945
Yes	155/618	(25.1%)	1.00		
No	12/47	(25.5%)	1.02	(0.52-2.02)	
Parent lived with partner at time of loss					0.002
Yes	111/497	(22.3%)	1.00		
No	56/164	(34.1%)	1.82	(1.24-2.68)	
Employment status at time of loss					<0.001
Not on sick-leave nor unemployed	130/580	(22.4%)	1.00		
Sick-leave	30/53	(56.6%)	4.58	(2.57-8.15)	
Unemployed	6/21	(28.6%)	1.40	(0.53-3.69)	
Residential area^c^					0.311
Rural	31/162	(19.1%)	1.00		
Town (population <10,000)	44/152	(28.9%)	1.70	(1.01-2.87)	
Small city (population <50,000)	33/128	(25.8%)	1.46	(0.84-2.54)	
Mid-sized city (population <200,000)	31/117	(26.5%)	1.58	(0.90-2.77)	
Larger city (population ≥200,000)	27/97	(27.8%)	1.65	(0.92-2.99)	
Educational level					0.262
University ≥ 3 years	36/159	(22.6%)	1.00		
University < 3 years	26/82	(31.7%)	1.60	(0.88-2.90)	
Secondary school	63/270	(23.3%)	1.02	(0.64-1.63)	
Elementary school or less	41/146	(28.1%)	1.36	(0.82-2.28)	
Belief in God					0.268
Yes	77/287	(26.8%)	1.00		
No	84/354	(23.7%)	0.82	(0.58-1.17)	
**Parent’s history of psychological stress and morbidity**					
History of psychological morbidity^d^					<0.001
No	84/502	(16.7%)	1.00		
Debuting during the last 10 years, before loss	31/67	(46.3%)	4.24	(2.49-7.24)	
Debuting more than 10 years ago	51/94	(54.3%)	5.85	(3.66-9.34)	
Loss of other significant person during 10 years prior to child’s suicide					0.173
No	68/298	(22.8%)	1.00		
Yes	97/358	(27.1%)	1.28	(0.90-1.83)	
History of suicide in other biological relatives					0.013
No	121/524	(23.1%)	1.00		
Yes	44/131	(33.6%)	1.69	(1.12-2.56)	
**Child’s background and parent-child relationship**					
Sex of deceased child					0.190
Son	109/461	(23.6%)	1.00		
Daughter	58/204	(28.4%)	1.28	(0.88-1.86)	
Age of deceased child					0.435
per 5 years (continuous)^a^			0.92	(0.74-1.14)	
Parent was deceased child’s legal guardian during upbringing					0.074
Yes	159/610	(26.1%)	1.00		
No	8/54	(14.8%)	0.49	(0.23-1.07)	
Biological parent					0.027
Yes	165/634	(26.0%)	1.00		
No^e^	2/31	(6.5%)	0.20	(0.05-0.83)	
Frequency of contact with child during child’s final year alive					0.090
Less than every month	8/37	(21.6%)	1.00		
At least every month	7/46	(15.2%)	0.65	(0.21-2.00)	
At least every week	60/268	(22.4%)	1.05	(0.45-2.41)	
Every day	91/313	(29.1%)	1.50	(0.66-3.41)	
Child lived with parent at time of loss^f^					0.249
No	88/390	(22.6%)	1.00		
Yes, part-time	24/84	(28.6%)	1.36	(0.80-2.30)	
Yes, full-time	54/190	(28.4%)	1.35	(0.91-2.00)	
**Child’s history of psychological stress and morbidity**					
Child had had contact with psychiatric clinic					0.995
Yes and admitted	58/226	(25.7%)	1.00		
Yes but not admitted	38/146	(26.0%)	1.03	(0.64-1.66)	
No	54/225	(24.0%)	0.97	(0.64-1.48)	
Do not know	15/60	(25.0%)	0.97	(0.50-1.86)	
Deliberate self-injury in child during final year alive					0.087
Yes, resulting in contact with healthcare services	34/119	(28.6%)	1.00		
Yes, with no contact with healthcare services	17/42	(40.5%)	1.76	(0.85-3.66)	
No	93/403	(23.1%)	0.78	(0.50-1.24)	
Do not know	21/92	(22.8%)	0.77	(0.41-1.43)	
Previous suicide attempts by child during final year alive^g^					0.889
No	94/383	(24.5%)	1.00		
Yes	37/150	(24.7%)	0.98	(0.64-1.52)	
Do not know	34/127	(26.8%)	1.11	(0.70-1.75)	
Previous suicide attempts by child earlier than final year					0.175
No	98/377	(26.0%)	1.00		
Yes	42/148	(28.4%)	1.11	(0.73-1.70)	
Do not know	25/135	(18.5%)	0.67	(0.41-1.09)	
**Child’s suicide**					
Method of suicide					0.162
Poisoning^h^	31/101	(30.7%)	1.00		
Violent means^i^	134/548	(24.5%)	0.72	(0.45-1.14)	
Parent witnessed body at site of suicide^j^					0.641
No	131/511	(25.6%)	1.00		
Yes	35/147	(23.8%)	0.90	(0.59-1.39)	
Parent viewed body in a formal setting^k^					0.737
Yes	118/459	(25.7%)	1.00		
No	49/202	(24.3%)	0.94	(0.64-1.37)	

^a^. Included with linear parametrization in this table for illustration purposes; subsequent multivariable modeling used non-linear parametrization.

^b^. Omitted from multivariable modeling due to high correlation with: country of birth of participant’s parents.

^c^. Current residential area at time of follow-up.

^d^. Psychological treatment, psychiatric diagnosis, or medication against anxiety, or low mood or depression, before the child’s suicide.

^e^. Includes 29 adoptive parents of children born in a developing country, and two adoptive parents of children born in Sweden.

^f^. Omitted from multivariable modeling due to high correlation with: frequency of contact with child during child’s final year alive.

^g^. Omitted from multivariable modeling due to high correlation with: previous suicide attempts by child earlier than final year.

^h^. Poisoning by e.g. medication, chemicals or gas.

^i^. Hanging or suffocation, in front of moving vehicle, jumping from a height, firearm discharge, self-induced motor vehicle crash, drowning, or cutting.

^j^. Found the child’s body, or saw the body at site of death shortly after someone else had found the body.

^k^. Viewed the child’s body after death in at least one of the hospital, the hospital church, a forensic medicine department, or at the funeral parlor.

**Fig 1:**
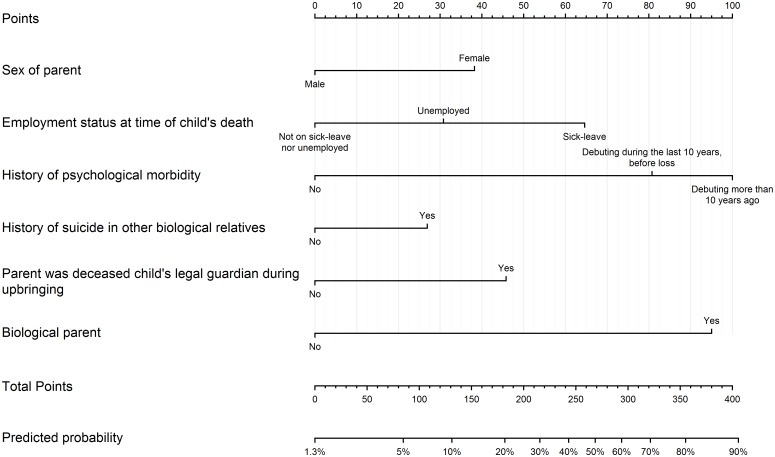
Reference nomogram for the multivariable prediction model. This nomogram is a graphical visualization of the multivariable model, where the model’s ORs have been rescaled to values between 0 and 100 points according to each variable’s lowest-risk category and the overall highest OR, respectively. To calculate the predicted probability of a bereaved parent to have moderate to severe depression two to six years after the loss, go through the questions and mark the values reflecting the parent’s circumstances. Read the corresponding number of points for each question from the top ruler (e.g. female sex gives 38 points), and sum the points from all questions. The predicted probability that the total sum of points represents can then be read from the two bottom rulers (e.g. a suicide-bereaved parent with a total of 85 points has a 5% model-predicted probability of depression).

The resulting multivariable prediction model had the following predictors: female sex, sick-leave or unemployment at time of loss, history of psychological morbidity, history of suicide in other biological relatives, if the parent had been the child’s legal guardian during their upbringing, and biological parenthood. None of the predictors had variance inflation factor above 1.1, indicating no problematic multicollinearity. The model is presented in Table 3, and in the form of a nomogram constructed from the model’s ORs in Fig 1 [23, 36], together with a comprehensive instruction in the figure legend on how to use the nomogram to obtain model-predicted probabilities of depression for individual bereaved parents.

**Table 3:** Odds ratios from the multivariable prediction model for long-term depression.

Variable	OR	(95% CI)	*p*-value
Sex			0.005
Male	1.00		
Female	1.84	(1.21-2.81)	
Employment status at time of loss			0.004
Not on sick-leave nor unemployed	1.00		
Sick-leave	2.81	(1.50-5.29)	
Unemployed	1.64	(0.56-4.75)	
History of psychological morbidity^a^			<0.001
No	1.00		
Debuting during the last 10 years, before loss	3.64	(2.09-6.34)	
Debuting more than 10 years ago	4.96	(3.00-8.21)	
History of suicide in other biological relatives			0.064
No	1.00		
Yes	1.54	(0.97-2.43)	
Parent was deceased child’s legal guardian during upbringing			0.104
Yes	1.00		
No	0.48	(0.20-1.16)	
Biological parent			0.046
Yes	1.00		
No^b^	0.22	(0.05-0.98)	

^a^. Psychological treatment, psychiatric diagnosis, or medication against anxiety, or low mood or depression, before the child’s suicide.

^b^. Includes 29 adoptive parents of children born in a developing country, and two adoptive parents of children born in Sweden.

### Internal validity

**Fig 2:**
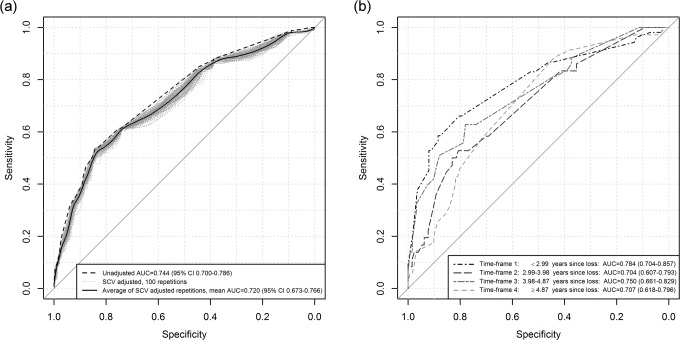
Classification performance. Receiver operating characteristic (ROC) curves, and corresponding areas under the curves (AUC) with 95% confidence intervals (CI), for (a) entire cohort, unadjusted and for 100 repetitions of ten-fold stratified cross-validation (SCV), and (b) for each of the four time-frames after cross-validation against a model derived from data in the other three time-frames. The ten-fold SCV adjusted values of AUC and CI limits are the corresponding mean values among the 100 repetitions, and the solid black line is a LOESS smoothed curve for the 100 SCV adjusted ROC curves outlined in gray.

**Fig 3:**
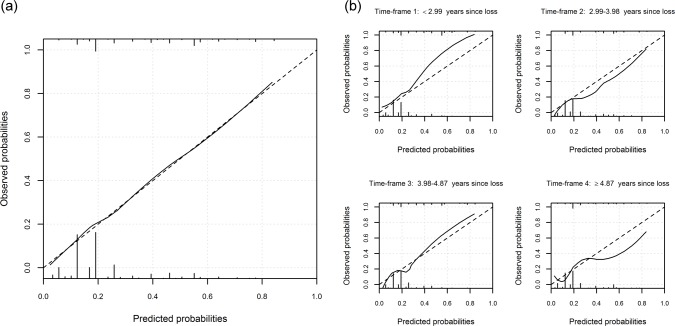
Calibration. Calibration plots between model-predicted and observed (LOESS smoothed) probabilities, for (a) entire cohort, and (b) in each time-frame. The histograms at top and bottom show the distribution of model-predicted probabilities among depressed and non-depressed respectively.

The model’s McFadden’s R^2^ was 0.220. Overall, the model had a SCV adjusted AUC of 0.720 (95% CI 0.673-0.766), and seemed well-calibrated across the whole range of predicted probabilities (Figs 2a and 3a).

As noted above, time since loss was associated with depression; when split into time-frames, prevalence of depression was 31.9%, 21.7%, 25.9% and 21.0%, respectively, in the earliest to the latest time-frame (logistic regression, linear term *p* = 0.036). We found the model to have slightly better classification performance in the earliest time-frame closest to loss, with AUC of 0.784 (95% CI 0.700-0.859), 0.704 (95% CI 0.611-0.797), 0.750 (95% CI 0.658-0.833) and 0.707 (95% CI 0.620-0.790) in time-frames 1, 2, 3 and 4, respectively (Fig 2b). Furthermore, calibration was found to be fair for probabilities up to about 20% in every time-frame, but for higher probabilities the model seemed to underestimate the observed probabilities in the earliest and overestimate them in the latest time-frame, with agreement being somewhat better in the two intermediate time-frames (Fig 3b).

### Post hoc and sensitivity analysis

We assessed associations between suicide in other biological relatives, having been the child’s legal guardian, and biological parenthood, with the contact between parent and child during the final year alive as well as the following current circumstances: whether the parent believes the suicide was triggered by a particular event, if the parent believes they could have prevented the suicide, and fear of death in other next-of-kin. Both suicide in other biological relatives and having been the child’s legal guardian during upbringing was found to have borderline non-significant correlations with fear of death in other next-of-kin (*γ* = +0.14, *p* = 0.083, and *γ* = +0.21, *p* = 0.073). Legal guardianship was furthermore strongly associated with the frequency of contact with the child during their last year alive (*γ* = +0.78, *p* < 0.001), corresponding to that 91% of legal guardians reported to have had contact with their child at least once a week versus 46% of non-guardians, whereas there was a negative correlation between biological parenthood and frequency of contact (*γ* = −0.53, *p* = 0.001).

As sensitivity analysis, we omitted the predictor biological parenthood and repeated the model-building procedure which resulted in a final model with the same predictors (apart from biological parenthood) as presented in Table 3, but as expected with a lower adjusted AUC of 0.713 instead of 0.720 (S1 Table and S1–S3 Figs).

## Discussion

We have presented a novel prediction model of long-term depression in parents who lost a son or daughter by suicide two to six years earlier. The model’s overall adjusted AUC of 0.720 (95% CI 0.673-0.765) reflects acceptable if not optimal internal classification performance [35], which may in turn imply that there are additional important predictors for depression beside those considered here. Psychological outcomes such as depression may be difficult to accurately predict due to the probable multitude of known and unknown predictors, such as biologic vulnerability, personality traits, stressful life events other than the suicide, and childhood maltreatment [28, 39]. We did not consider other possibly important post-loss predictors such as social support, or appraisal and coping [28]. In order to enable use of our prediction model in clinical practice, we chose to focus on formal circumstances more readily assessable at the time of loss, and to avoid using predictors concerning later circumstances not known at that time.

We found the model’s predicted probabilities to be fairly calibrated in both early and late time-frames for the half of parents with characteristics that reflect up to about 20% risk of depression, but for those at higher risk it seems to underestimate the true risk for those who had a recent loss and overestimate the risk for those with long time since loss. This is consistent with an overall decrease in the risk of depression with longer time since loss. As evidenced by the corresponding AUCs the model’s ability to rank individuals from lowest to highest risk proved acceptable in all four time-frames, although we found that classification performance was best in the earliest time-frame close to the loss. This might reflect that the considered predictors were chosen to describe circumstances at the time of loss, some of which (e.g. employment status) may change over time and thus become less relevant as time passes from the occasion when they were assessed.

A history of psychological morbidity at the time of loss was among the most prominent predictors identified, both in terms of the strength of association and prevalence; 24% reported premorbidity which had debuted at some time before the loss, among which the 14% whose morbidity had debuted more than 10 years earlier seemed particularly vulnerable. While we lack data on the entire life course of depression among participants, it is expected that individuals with prior morbidity have a higher risk of depression after the loss, given the high chronicity of depression [40], its overall high recurrence rates [41], as well as the available evidence on stressful life events as triggers for depression recurrence [41].

Female sex and unemployment are well-established predictors of depression in the general population [42, 43]. Associations between sick-leave and psychological morbidity have also been reported, to a lesser extent among those on sick-leave due to somatic causes, and in particular among sickness-absentees where psychological morbidity is the cause of sick-leave [44].

The other three of our included predictors may hypothetically be related to more complex phenomena of familial and biological patterns, as well as worry for other family members, self-blame and feelings of guilt. We have previously reported that as compared to a matched sample from the general population (not considered in the present investigation), our study population has very similar proportions reporting a history of psychological morbidity which debuted more than 10 years earlier (14% vs 14%) [3]. The higher risk of depression among parents with a history of suicide in other biological relatives, as well as among biological parents, on the other hand seem in line with previous reports of familial patterns in predisposition for psychological morbidity among families of suicide victims [2, 4, 20]. Neither finding is however inconsistent with there being a minority subgroup among suicide-victims with a predominantly familial component, since we have little information on the severity of the morbidity among the suicide-bereaved parents who report a history of psychological morbidity. Then again, our results also indicate that those who have experienced suicide in other biological relatives might be somewhat more inclined to worry about death in other next-of-kin, which points to the possibility that the effect of suicide in other biological relatives might in part be a consequence of higher stress.

Furthermore, the Multigeneration Register from which participants were identified only records non-biological parents to whom parenthood has been transferred though a formal adoption. Adoption is typically a planned parenthood in a stable relationship where the prospective parents have been required by authorities to show sufficient psychological, physical and economic stability, so it is possible that non-biological parenthood may simply be indicative of favorable circumstances among adoptive parents associated with lower disposition to psychological morbidity. Also, adopted children may be traumatized, and a higher risk of psychological morbidity and suicide is established among adoptees [45], especially among those whose biological parents have a history of mental illness or suicide [46]. Perhaps such prior circumstances outside the parents’ control may serve as an external explanation of the child’s suicide for some bereaved parents, thus helping adoptive parents to avoid placing blame on themselves.

The lower risk seen in parents who were not the child’s legal guardian during upbringing may, based on our further findings of correlation with frequency of contact, possibly be related to the strength of bond between parent and child, and to a related lower propensity for placing the blame for the child’s suicide on oneself. Our univariable results give partial support to the former, in that the risk of depression was somewhat higher for those who had contact with their child every day during the child’s final year alive.

Noteworthy negative findings were that none of the considered formal circumstances related to the suicide was found to be associated with depression. We have previously reported on the lack of association with moderate to severe depression for some of these suicide-related predictors [47, 48], although a weak association seems to exist between viewing the body in a formal setting and the full PHQ-9 sum [47]. It is possible that there are further important aspects of a suicide besides those that we have covered, but it may also reflect that a child’s suicide is a significant trauma in itself regardless of the formal circumstances surrounding the event.

In two recent Dutch studies, other prediction models for depression among bereaved groups have been proposed. Based on a longitudinal cohort of 153 suicide-bereaved spouses and first-degree relatives (primarily parents, children and siblings) followed at 2.5, 13 and 96-120 months post-loss, de Groot and Kollen [18] proposed separate prediction models for depression, complicated grief, and suicide ideation. No measures of overall model fit were presented, complicating comparison with our model. Contrary to our results the authors did not find history of psychological morbidity to affect depression following their multivariable model selection. Their model included measures of current post-loss psychological morbidity, time since loss and help-seeking as predictors. Wijngaards-de Meij and coworkers [24] presented prediction models for depression and grief based on a longitudinal follow-up of 219 parent couples 6 to 20 months after bereavement by the death of a child by any cause. The depression model had a proportion variance explained of 0.30, which is somewhat higher than our model’s proportion variance explained (McFadden’s R^2^) of 0.220. This may in part be by design, as they modeled repeated measures of a quantitative score as depression outcome while our outcome was a single dichotomous measure, but of note is that the model included post-loss predictors such as time since loss, professional help-seeking and new pregnancy which may be expected to improve post-loss prediction of concurrent depression, but limits its utility in clinical practice at the time of loss.

Our study has a number of strengths and limitations. The major strength is the population-based approach where we have tried to contact all parents in the Swedish population who fulfilled our inclusion criteria. Another is the high response-rate which speaks in favor of the generalizability of the findings. One limitation is that, due to the anonymous participation, we lack data on which participants were parents of the same deceased child; potentially this could lead to lower variability in the data as compared to if all responses were independent of each other. The modeled outcome is a questionnaire-based proxy measurement of depression which may not reflect a depression diagnosis. We have chosen to focus on potential predictors which we deem resilient to recall bias, but cannot rule out its existence. The lack of longitudinal data is a limitation when the studied outcome varies with time; our findings however indicate acceptable performance at least for those at up to 20% risk of depression in all of the considered time-frames. Prevalence of non-biological parenthood is relatively low and corresponding point estimates are thus based on a small sample size. However, omitting the predictor biological parenthood resulted in a slightly lower AUC and would give a model that exaggerates the risk of depression among non-biological parents as no other predictor was found in its place.

Depression is common after the loss of a family member by unnatural causes [1, 6]. Most suicide-bereaved parents will come into contact with health services pertaining to the death of their child. Yet, in our study two out of five parents indicated to be depressed two to six years following their child’s suicide were without pharmaceutical treatment, despite its reported efficacy in treating bereavement-related depression [11]. It seems crucial that tools are developed to help health-care professionals predict which individuals are at high risk of post-loss morbidity, based on what is known at the time of loss. Our proposed prediction model shows promising internal validity, however the performance of any prediction model should be validated and tested with new data before use, especially if the intended use is in a population outside the one in which the model was developed. While no statistical prediction model may substitute an individual clinical assessment, in the longer run similar methodology as demonstrated here could be used to create clinical prediction tools to aid in individual patient and next-of-kin contacts. Our results however underline the importance for healthcare providers to be aware of psychological premorbidity in suicide-bereaved parents, as this seems to be the most prominent of our considered predictors for long-term depression.

## Supporting Information

S1 Appendix Model development details. (PDF)

S1 Table Sensitivity analysis results following omission of the predictor biological parenthood: Odds ratios from the reduced multivariable prediction model for long-term depression. (PDF)

S1 Fig Sensitivity analysis results following omission of the predictor biological parenthood: Reference nomogram for the reduced multivariable prediction model. (TIF)

S2 Fig Sensitivity analysis results following omission of the predictor biological parenthood: Classification performance of the reduced multivariable prediction model. Receiver operating characteristic (ROC) curves, and corresponding areas under the curves (AUC) with 95% confidence intervals (CI), for (a) entire cohort, unadjusted and for 100 repetitions of ten-fold stratified cross-validation (SCV), and (b) for each of the four time-frames after cross-validation against a model derived from data in the other three time-frames. The ten-fold SCV adjusted values of AUC and CI limits are the corresponding mean values among the 100 repetitions, and the solid black line is a LOESS smoothed curve for the 100 SCV adjusted ROC curves outlined in gray.
(TIF)

S3 Fig Sensitivity analysis results following omission of the predictor biological parenthood: Calibration of the reduced multivariable prediction model. Calibration plots between model-predicted and observed (LOESS smoothed) probabilities, for (a) entire cohort, and (b) in each time-frame. The histograms at top and bottom show the distribution of model-predicted probabilities among depressed and non-depressed respectively.
(TIF)

S1 File Dataset with all variables considered for the multivariable prediction model for the n = 665 included parents. (TXT)
