# Supplementary material for: Depression among Parents Two to Six Years Following the Loss of a Child by Suicide: A Novel Prediction Model
Source: PLoS One. 2016 Oct 3;11(10):e0164091. doi: 10.1371/journal.pone.0164091 (PMC5047457; doi:10.1371/journal.pone.0164091)
Supplement: S1 Appendix — (PDF) [file pone.0164091.s001.pdf]

S1 Appendix: Model development details

To avoid losing individuals due to missing data, we employed a *k*-nearest-neighbor imputation with *k*=7. Gower distance was used to select neighbors since it is applicable to datasets which have a mix of nominal, ordinal and continuous variables. The resulting imputation-completed data were used for all modeling, whereas we present the original incomplete data for all descriptives.

We used logistic regression for all modeling, and present odds ratios (OR) with 95% confidence intervals (CI). Initially we considered a number of closely related preliminary univariable models in order to select the list of variables to subsequently consider for multivariable modeling. To assess non-linearity and select how to model the effect of continuous variables, we formed preliminary models including the variables as linear terms or as restricted cubic splines with 3, 4 or 5 knots. The parametrization among those considered which minimized the Akaike information criterion (AIC) was used for all subsequent modeling.

> **Result.** For parent's age we found that a restricted cubic splines fit with 3 knots minimized the AIC. For child's age a linear parametrization had the lowest AIC, and thus these parametrizations were used henceforth. However neither variable were later included in the multivariable model.

To avoid problems related to multicollinearity, i.e. strong correlation between considered predictors, we calculated pairwise correlations between our 26 considered model covariates (using dummy variable expansion for categorical variables). For pairs of covariates which showed a Pearson correlation ≥ 0.5 we created and compared the corresponding univariable models, and selected only one variable to retain out of each pair based on AIC.

> **Result.** Variables found to have a pairwise correlation ≥ 0.5 were the following, with the omitted variable in each pair in brackets: [country of birth of parent], and of parent's own parents; frequency of contact, and [whether the child lived with the parent at the time of the suicide]; and whether the child had made previous suicide-attempts during the last year alive, and [earlier than that].

Following this, we created a multivariable model, and based variable selection on difference between observed outcome and model-predicted probabilities as measured by the model deviance (-2 $\times$ the log-likelihood). To reduce the number of possible models (and thus computational resources), in a first round we only considered variables which had $p < 0.25$ in univariable logistic regression. Among those

variables we performed an all subsets selection and considered models with all possible combinations of main effects. Since the model deviance does not penalize unnecessary model complexity, to avoid overfitting we used ten-fold stratified cross-validation (SCV), and to reduce the impact of random chance in the construction of cross-validation folds, the all subsets selection was repeated on multiple refolds of the data. The model which minimized the mean of the resulting SCV adjusted model deviances was selected as a first model. Initially a total of 100 refolds were used; this number was then increased to accommodate the differences in mean deviance between models as compared to the corresponding standard errors: we re-evaluated the 100 previously best-performing models in 5,000 new refolds of the data to ensure that the mean SCV adjusted deviance of the best-performing model differed at least 3 standard errors from the second best-performing model.

> **Result.** In the first 100 refolds as well as after the further 5,000 refolds, the same preliminary model was identified, with predictors: female sex, sick-leave or unemployment at time of loss, history of psychological morbidity, history of suicide in other biological relatives, if the parent had been the child's legal guardian during their upbringing, and biological parenthood. After 100 refolds the difference in mean deviance between the first and second and between the first and 100th best performing models were 0.1 and 4.0 standard errors respectively. After the 5,000 further refolds the mean SCV adjusted model deviance of the best performing model differed 4.9 standard errors from that of the second best performing model.

In a second round, we reconsidered the initially omitted variables with univariable $p \geq 0.25$ and compared all models created with the variables selected in the first round plus all combinations of these additional variables, initially in 100 refolds followed by 2,000 confirmatory refolds.

> **Result.** Neither of the additional variables was found to reduce the model deviance so neither was included. The difference in mean deviance between the preliminary model from the first round and the best performing model among those in round two were 0.8 standard errors after 100 refolds, and after 2,000 refolds the difference was 3.4 standard errors.

In a third round, we considered interactions between the variables in our preliminary model: we tried to include all possible two-way interactions one at a time and assess improvement in SCV adjusted deviance. Interaction terms which caused complete separation of data in at least one refold were dismissed. Initially we used 100 refolds, followed by 200 new refolds. We continued this process until no further interaction term was found to improve the cross-validated deviance, thus yielding our final model.

Result. Interaction terms involving variables Legal guardian or Biological parenthood which both have rare categories frequently resulted in complete separation (empty cells) and were therefore omitted. Neither of the remaining interaction terms was found to reduce the mean model deviance so neither was included; minimum difference in mean deviance between the main effects model and models involving a two-way interaction was 2.5 standard errors after 100 refolds, and 3.6 standard errors after 200 refolds. The main effects model identified in the first round was thus retained as the final model.
