# Supplementary material for: Depression among Parents Two to Six Years Following the Loss of a Child by Suicide: A Novel Prediction Model
Source: PLoS One. 2016 Oct 3;11(10):e0164091. doi: 10.1371/journal.pone.0164091 (PMC5047457; doi:10.1371/journal.pone.0164091)
Supplement: S1 Table — (PDF) [file pone.0164091.s002.pdf]

S1 Table: Sensitivity analysis results following omission of the predictor biological parenthood: Odds ratios from the reduced multivariable prediction model for long-term depression

Variable	Category	OR	(95% CI)	*p*-value
Sex				0.004
	Male	1.00		
	Female	1.85	(1.22-2.82)	
Employment status at time of loss				0.004
	Not on sick-leave nor unemployed	1.00		
	Sick-leave	2.79	(1.49-5.23)	
	Unemployed	1.63	(0.57-4.71)	
History of psychological morbidity[a]				<0.001
	No	1.00		
	Debuting during the last 10 years, before loss	3.60	(2.07-6.25)	
	Debuting more than 10 years ago	5.10	(3.09-8.42)	
History of suicide in other biological relatives				0.051
	No	1.00		
	Yes	1.57	(1.00-2.47)	
Parent was deceased child's legal guardian during upbringing				0.100
	Yes	1.00		
	No	0.48	(0.20-1.15)	

[a] Psychological treatment, psychiatric diagnosis, or medication against anxiety, or low mood or depression, before the child's suicide.
