# Supplementary figures and images for: Depression among Parents Two to Six Years Following the Loss of a Child by Suicide: A Novel Prediction Model
Source: PLoS One. 2016 Oct 3;11(10):e0164091. doi: 10.1371/journal.pone.0164091 (PMC5047457; doi:10.1371/journal.pone.0164091)

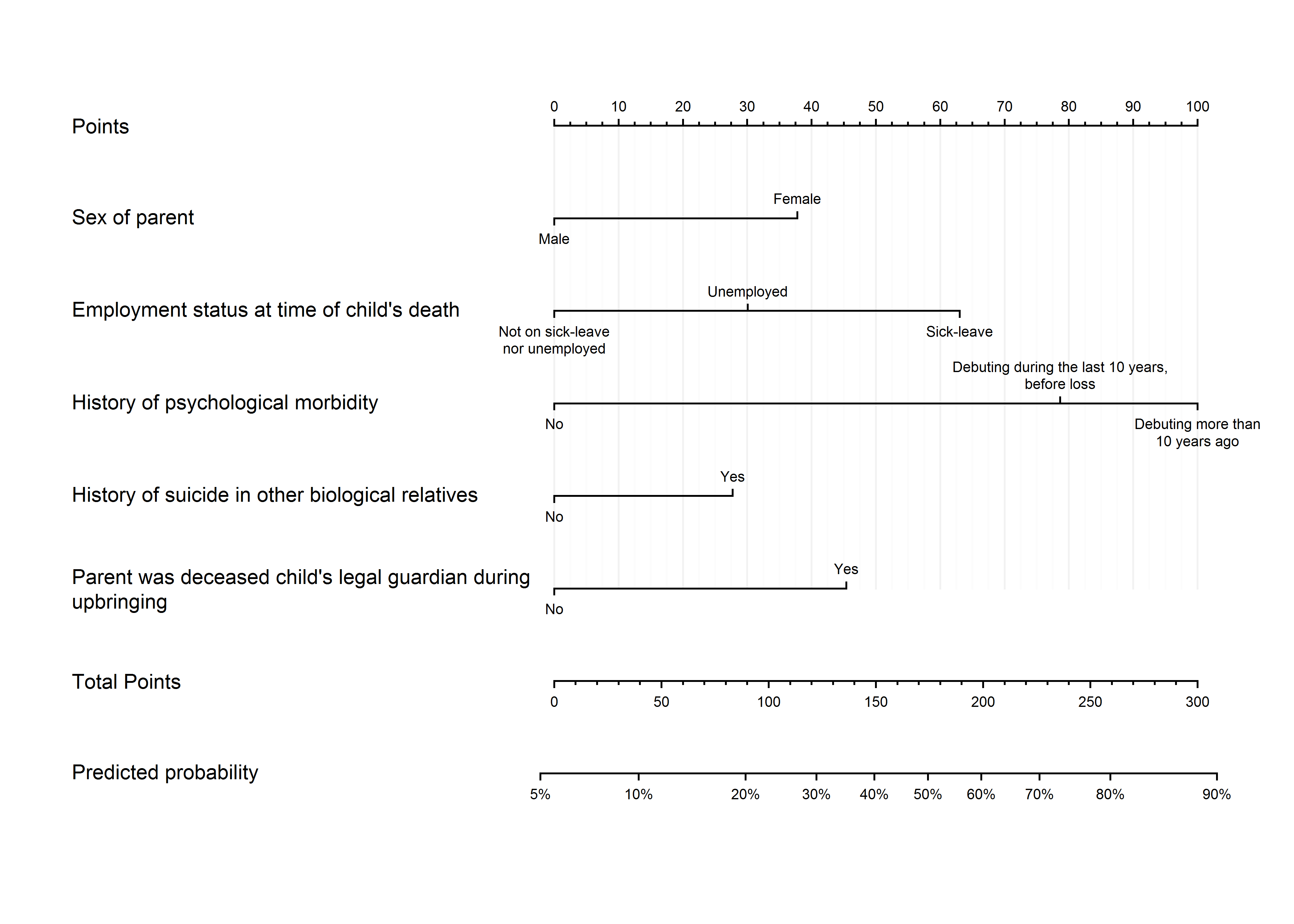

Supplement: S1 Fig — (TIF) [file pone.0164091.s003.tif]

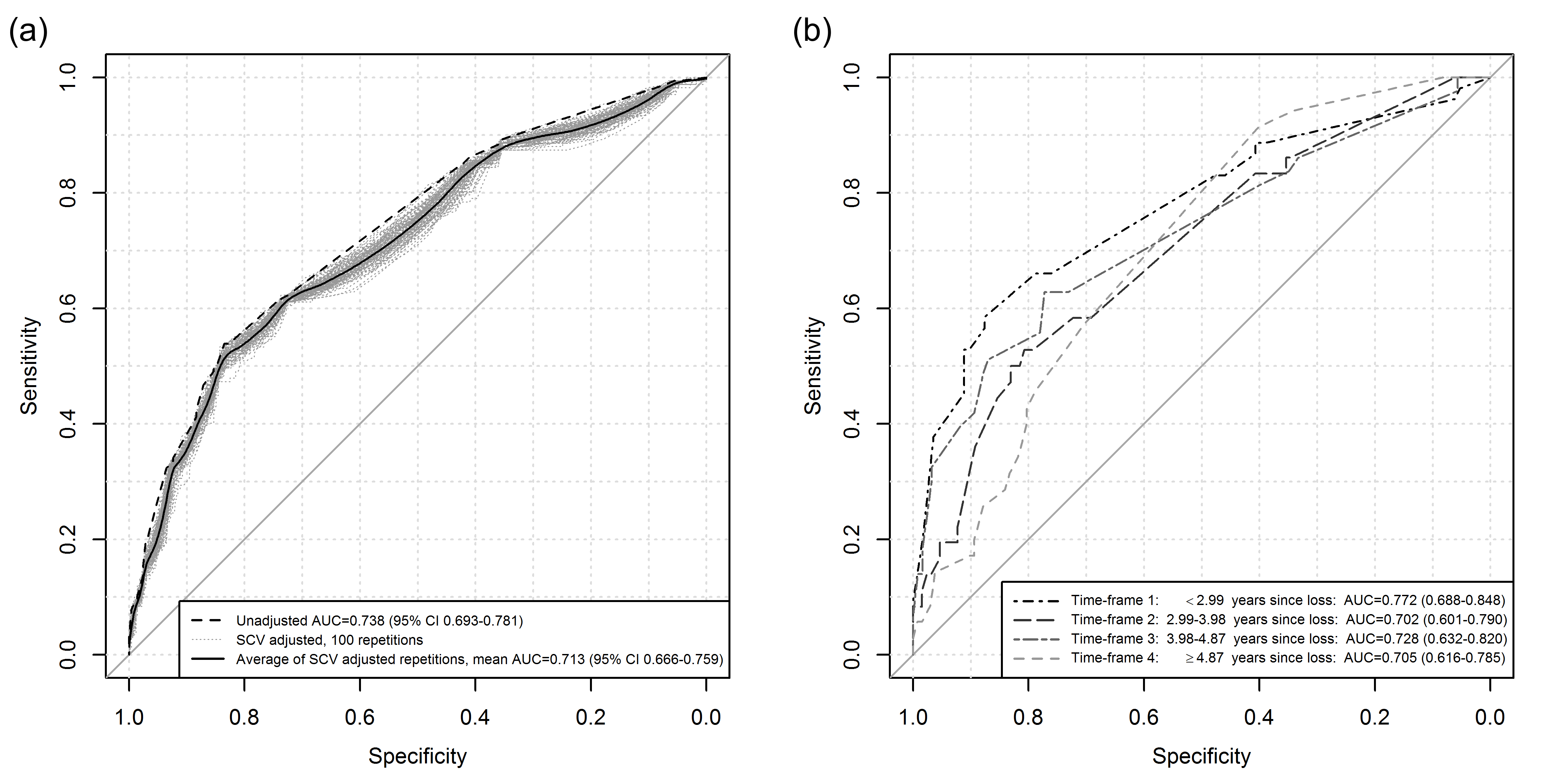

Supplement: S2 Fig — Receiver operating characteristic (ROC) curves, and corresponding areas under the curves (AUC) with 95% confidence intervals (CI), for (a) entire cohort, unadjusted and for 100 repetitions of ten-fold stratified cross-validation (SCV), and (b) for each of the four time-frames after cross-validation against a model derived from data in the other three time-frames. The ten-fold SCV adjusted values of AUC and CI limits are the corresponding mean values among the 100 repetitions, and the solid black line is a LOESS smoothed curve for the 100 SCV adjusted ROC curves outlined in gray. (TIF) [file pone.0164091.s004.tif]

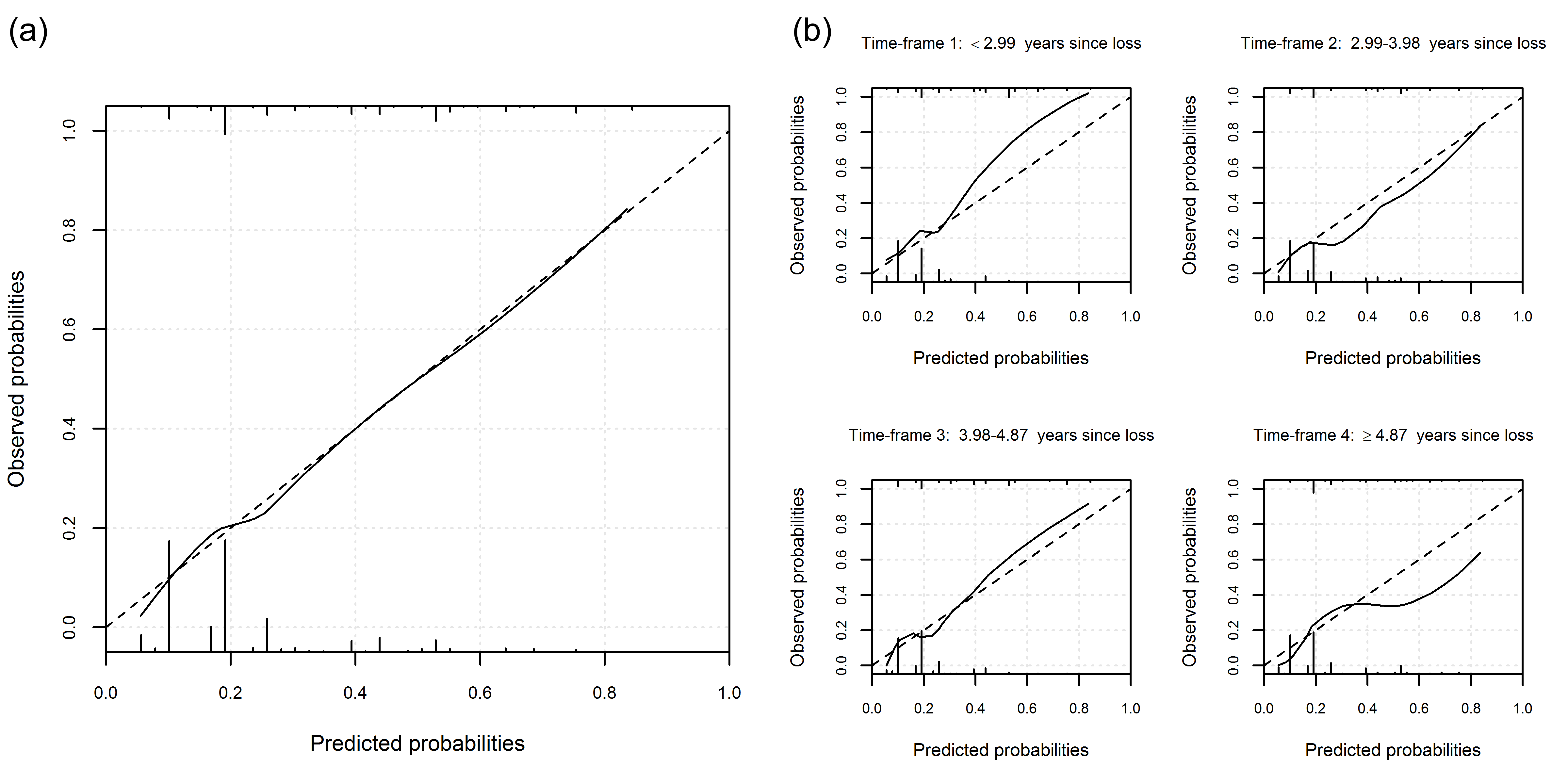

Supplement: S3 Fig — Calibration plots between model-predicted and observed (LOESS smoothed) probabilities, for (a) entire cohort, and (b) in each time-frame. The histograms at top and bottom show the distribution of model-predicted probabilities among depressed and non-depressed respectively. (TIF) [file pone.0164091.s005.tif]
